# Interobserver Reliability of Tongue Diagnosis Using Traditional Korean Medicine for Stroke Patients

**DOI:** 10.1155/2012/209345

**Published:** 2012-02-28

**Authors:** Mi Mi Ko, Ju Ah Lee, Byoung-Kab Kang, Tae-Yong Park, Jungsup Lee, Myeong Soo Lee

**Affiliations:** ^1^Medical Research Division, Korea Institute of Oriental Medicine, 1672 Yuseongdae-ro, Yuseong-gu, Daejeon 305-811, Republic of Korea; ^2^Department of Oriental Rehabilitation Medicine, Korean National Rehabilitation Center, 58, Samgaksan-ro, Gangbuk-Gu, Seoul 142-884, Republic of Korea

## Abstract

Observation of the tongue, also known as tongue diagnosis, is an important procedure in diagnosis by inspection in Traditional Korean medicine (TKM). We investigated the reliability of TKM tongue diagnosis in stroke patients by evaluating interobserver reliability regarding tongue indicators as part of the project named the Fundamental Study for the Standardization and Objectification of Pattern Identification in TKM for Stroke (SOPI-Stroke). A total of 658 patients with stroke admitted to 9 oriental medical university hospitals participated. Each patient was independently seen by two experts from the same department for an examination of the status of the tongue. Interobserver agreement about subjects regarding pattern identification with the same opinion between the raters (*n* = 451) was generally high, ranging from “moderate” to “excellent”. Interobserver agreement was nearly perfect for certain signs of special tongue appearance (mirror, spotted, and bluish purple), poor for one of the tongue colors (pale) and moderate for others. Clinicians displayed measurable agreement regarding tongue indicators via both observation and pattern identification consistency. However, interobserver reliability regarding tongue color and fur quality was relatively low. Therefore, it is necessary to improve objectivity and reproducibility of tongue diagnosis through the development of detail-oriented criteria and enhanced training of clinicians.

## 1. Introduction

Stroke is the second most common cause of death in developed countries and thus is a major health problem [1]. According to the 2009 Annual Public Health Report by the Korean National Statistical Office, cerebrovascular disease, or stroke, was the second-leading cause of disease-related deaths in Korea, after cancer [2]. In Korea, many stroke patients receive traditional medical care because the country has its own system of traditional alternative medicine called Traditional korean medicine (TKM), the role of which has been emphasized in stroke management [3].

The Korean medical diagnosis system has unique characteristics similar to the traditional Chinese medical diagnosis system. One such feature is pattern identification (PI), which is based on information obtained from four diagnostic processes including inspection, listening and smelling, inquiry, and palpation [4]. PI is a diagnostic system that entails a comprehensive analysis of symptoms and signs, with implications for determining the cause, nature, and location of the illness, the patient's physical condition, and the patient's treatment [3, 5]. The inspection process involves the examination of the patient's symptoms or disease by observing his or her shape, expression, and tongue [6], among others. Observation of the tongue, also known as tongue diagnosis, is an important procedure in diagnosis by inspection in TKM. The status of the tongue is an important indicator in the diagnosis of one's condition, including the physiological and clinicopathological changes of internal organs in the body [7]. A number of studies have shown that tongue diagnosis plays an important role in clinical prognosis and treatment [8–15], specifically in patients with a history of stroke. However, the clinical competence of tongue diagnosis was determined by the experience and knowledge of the clinicians who used tongue diagnosis. Environmental factors, such as differences between light sources and levels of brightness also had significant influences on clinicians and their diagnostic decisions using the tongue. Unfortunately, much of the experiences in traditional tongue diagnosis have not been verified scientifically or quantitatively. Therefore, it is necessary to build an objective diagnostic standard for tongue diagnosis [7]. We investigated the reliability of TKM tongue diagnosis in stroke patients by evaluating interobserver reliability regarding tongue indicators as achieved by TKM practitioners.

## 2. Methods

### 2.1. Study Subjects

**Figure 1:**
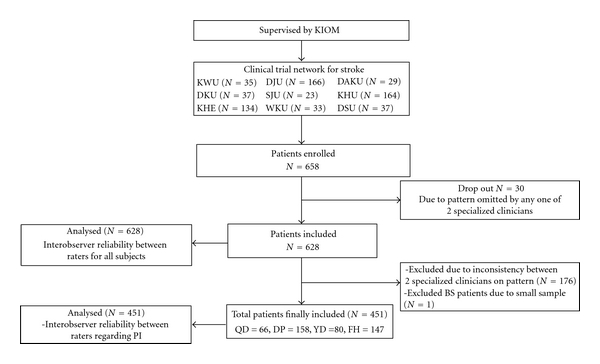
Flow chart showing patient enrollment in study. KIOM: Korean Institute of Oriental Medicine; KWU: Kyung Won Oriental Medical Hospital; KHE: Kyung Hee East-West Neo Medical Center; DJU: Dae Jeon Oriental Medical Hospital; DAKU: Dae Gu Hanny University Medical Center; DKU: Dong Guk International Hospital; SJU: Sang Ji Oriental Medical Hospital; KHU: Kyung Hee Oriental Medical Center; WKU: Won Kwang Oriental Medical Hospital; DSU: Dong Sin Oriental Medical Hospital; PI: pattern identification; QD: qi deficiency pattern; DP: dampness-phlegm pattern; YD: yin deficiency pattern; FH: fire-heat pattern; BS: blood stasis pattern.

The data for this analysis were collected as part of the project named the Fundamental Study for the Standardization and Objectification of Pattern Identification in TKM for Stroke (SOPI-Stroke). Stroke patients admitted to the following oriental medical university hospitals, Kyung Hee Oriental Medical Center (Seoul), Kyung Hee East-West Neo Medical Center (Seoul), Dong Guk International Hospital (Kyunggi-do), Kyung Won Oriental Medical Hospital (Incheon), Dae Jeon Oriental Medical Hospital (Daejeon), Dong Sin Oriental Medical Hospital (Gwangju), Won Kwang Oriental Medical Hospital (Jeollabuk-do), Dae Gu Hanny University Medical Center (Daegu), and Sang Ji Oriental Medical Hospital (Gangwon-do), participated in this study between February 2010 and December 2010 (Figure 1). All patients provided written informed consent under procedures approved by institutional review boards (IRB). Eligibility inclusion criteria were that participants had to be enrolled within 30 days of the onset of their symptoms as confirmed by imaging diagnosis, such as computerized tomography (CT) or magnetic resonance imaging (MRI). Exclusion criteria were traumatic stroke patients, such as those with subarachnoid, subdural, or epidural hemorrhage.

### 2.2. Data Processing and Analysis

All patients were seen by two experts from the same department in each site, who were well trained in standard operation procedures (SOPs) [Appendix] and were subjected to an examination of the status of the tongue, tongue color (pale, red, and bluish purple), fur color (white fur, yellow fur), fur quality (thick fur, thin fur, moist fur, and dry fur), and special tongue appearance (teeth marked, enlarged, mirror, and spotted). The examination parameters were extracted from parts of a case report form (CRF) for the standardization of stroke diagnosis that had been developed by an expert committee organized by the Korean Institute of Oriental Medicine (KIOM). These assessments were given individually without discussions among the clinicians. Descriptions of the grading severity for each variable were scored as the following: 1 = very much so, 2 = Much so, and 3 = Not so much. In particular, the clinicians had to measure the stroke PI of each patient following the fire-heat pattern, the phlegm-dampness pattern, the blood stasis pattern, the qi deficiency pattern, and the Yin deficiency pattern, as suggested by the KIOM [3, 16–18].

Interobserver reliability was measured in three ways, using simple percentage agreements, Cohen's kappa coefficient and Gwet's AC_1_ statistic, as well as via the corresponding confidence intervals (CI). Kappa, the preferred measure of rater reliability for nominal data, measures the reliability of agreement between two or more independent raters using a rating scheme with mutually exclusive categories. In general, definitive kappa interpretations have been proposed [19–24]. For most purposes, however, values ≤0.40 represent poor agreement, values between 0.40 and 0.75 represent moderate to good agreement, and values ≥0.75 indicate excellent agreement [24]. The AC_1_ statistic is not vulnerable to the well-known paradoxes that make Kappa appear ineffective [25–27]. First, interobserver reliability for the tongue indicator among all subjects was calculated via simple percentage agreements, Cohen's kappa coefficient, and Gwet's AC_1_ statistic. Later, interobserver reliability regarding PI with same opinions between the raters was calculated in the same way. The blood stasis pattern was omitted because the sample size was too small (*n* = 1). The data were statistically analyzed with SAS software, version 9.1.3 (SAS Institute Inc., Cary, NC).

### 2.3. Ethical Approval

This study was approved by institutional review board of the KIOM and by each of the oriental medical university hospitals.

## 3. Results

A total of 658 stroke patients were enrolled in the study. Thirty patients were excluded from analysis due to PI omitted by any one of 2 TKM clinicians. The interobserver reliability results regarding tongue indicators for all subjects (*n* = 628) are shown in Table 1. The kappa measure of agreement between the two experts was generally moderate to good for the tongue indicators, ranging from 0.42 to 0.69, except for moist fur (*κ* = 0.29) and spotted (*κ* = 0.37). Moreover, the AC_1_ measure of agreement between the two experts was generally high for the tongue indicators, ranging from “moderate” (AC_1_ = 0.43) to “excellent” (AC_1_ = 0.97). Agreement, as assessed by the kappa values, was considerably lower than the AC_1_ values in most cases.

The results of interobserver reliability for subjects of PI with the same opinion between the raters are shown in Table 2. A total of 451 stroke patients received PI with the following same resulting opinions by the raters: Fire-Heat Pattern (*n* = 147), Phlegm-Dampness Pattern (*n* = 158), Yin Deficiency Pattern (*n* = 80), and Qi Deficiency Pattern (*n* = 66). The blood stasis pattern was excluded because the sample size was too small (*n* = 1). 

The kappa measure of agreement for the subjects of PI was generally moderate to good for the tongue indicators, ranging from 0.40 to 0.72, except for moist fur (*κ* = 0.31). Moreover, the AC_1_ measure of agreement between the two experts was generally high for the tongue indicators, ranging from “moderate” (AC_1_ = 0.5) to “excellent” (AC_1_ = 0.98) (Table 2).

## 4. Discussion

Inspection of the tongue in TKM diagnosis, as well as in western medicine [28], is one of the most important approaches for obtaining significant evidence in diagnosing the patient's health conditions [7]. It is used to observe the color, coating, and body of the tongue, among other features, in rendering a disease diagnosis. 

Also, as tongue diagnosis has played a prominent role in the diagnosis and subsequent treatment of stroke patients, it has attracted an increasing amount of attention in oriental medicine [8–15]. Park et al. [12] analyzed markers that classified tongue body color, fur, fur quality, dryness, and shape to standardize tongue diagnosis and PI for stroke patients. Choi et al. [14], to assess the usefulness of tongue diagnosis in evaluating PI, observed the coating of the tongue and compared it with PI in acute stroke stage patients within 72 hours from the onset of stroke. In his study, a red tongue was significantly related to the fire-heat pattern and the yin deficiency pattern, while a faint white tongue was related to the phlegm-dampness pattern. Thin fur was related to the Wind and fire-heat pattern, and thick fur was related to the phlegm-dampness and blood stasis patterns. Another study [15] by the same author found that a stroke patient's motor recovery might be related to tongue diagnosis. In Kim et al.'s recent study [3], the authors attempted to standardize the oriental medical PI for stroke patients using logistic regressions. An interesting finding was that all of the patterns in their study basically included tongue and pulse diagnoses in their final equations. This result shows that TKM clinicians tongue and pulse diagnosis are seriously considered in their patient management.

However, traditional tongue diagnosis does have its inevitable limitations because the clinical skill involved in tongue diagnosis depends on the clinician's experience and knowledge as well as on environmental factors that can exert a significant influence on the diagnostic results. Therefore, it is necessary to build an objective diagnostic standard for tongue diagnosis. To date, only a few studies have reached wide consensus among TKM clinicians, while many studies have investigated agreement measures for western medical diagnosis [29]. 

An evaluation of interobserver reliability is important when one is interested in the “true” differences among observers that often report different values for the same quantity. In other words, interobserver reliability, rather than the total observer reliability, should be used to explore the causes of the disagreements among observers. The total observer reliability masks these sources of disagreement because it contains both interobserver reliability (true differences) and intraobserver reliability (random error among the observations) made by the same observer for the same subject [30]. Li et al. [31] used the kappa value to evaluate the consistency of tongue and pulse signs for 55 patients as observed by traditional Chinese medicine (TCM) clinicians. Zhang et al. [32] analyzed the effect of training on improved agreement in TCM diagnosis among its practitioners based on a sample of 42 patients with rheumatoid arthritis (RA). Mist et al. [33], using interrater correlations and kappa values, assessed whether a training process that focused on a questionnaire-based diagnosis would improve agreement in traditional Chinese medicine TCM diagnosis. Finally, Kim et al. [34] examined the reliability of TCM tongue inspection by evaluating inter- and intrapractitioner agreement levels for specific tongue characteristics. 

The data for this analysis were collected as parts of a multicenter study of standardization of stroke diagnosis in Korea. In this study, the evaluation of interobserver reliability in tongue status in stroke patients, as achieved by TKM clinicians, as well as interobserver reliability in all subjects (or subjects of PI with same the opinions between the raters), was calculated as simple percentage agreements, kappa values and AC_1_ measures. When investigating agreement between observers, clinicians have long used kappa and other chance-adjusted measures. A commonly used scale used to interpret kappa derives from the work of Landis and Koch in 1977 [20]. However, the appropriateness of kappa as a measure of agreement has recently been debated [26, 27]. A relatively new statistic, the AC_1_, has been suggested by Gwet to adjust for chance in agreement studies [25, 35]. 

According to our results, interobserver agreement in tongue diagnosis between the raters was generally moderate to good. The AC_1_ measure of agreement between the two experts was generally moderate to good for the tongue indicators, ranging from 0.43 to 0.97. 

In particular, the AC_1_ measure of agreement was nearly perfect in mirror, spotted, and bluish purple tongue. These tongue indicators are certain signs of special tongue appearance. Mirror tongue means that the surface of tongue is smooth and shiny like a mirror, without fur. Spotted tongue means that there are purple spots on the whole tongue and bluish purple tongue means that the color of tongue body is bluish purple, or bluish purple spots appear on the surface of the tongue. It is thought that a description of these indicators is relatively objective, that agreement is very high. Whereas the AC_1_ of pale, thin fur, moist fur is lower than 0.5. The reason why the AC_1_ of these indicators is low is that perception of quality and color of tongue vary markedly from person to person. Therefore, it is necessary to improve the validity and reliability of tongue diagnosis through the development of detail-oriented criteria and enhanced training of clinicians.

One limitation of our study is the fact that we did not analyze the impact of the each site participated in this study. All patients were allocated into two experts among the eighteen clinicians in each site. While the large number of clinicians who participated in the study increased the generalizability of the results, it is possible that the variety of experiences offered by these clinicians was another limitation to the study. All clinicians who had at least more than three years of clinical experience in the field took regular SOPs training twice a year, therefore we assumed all clinicians have equal ability to take information from the patients. But, in reality it is certain that this assumption is not true. We will consider the subject carefully in the future work. Furthermore, this study has a limitation in that the actual diagnosis process in TKM is carried out not only through tongue diagnosis but also through other three diagnostic processes. Further studies may be necessary to conduct a comprehensive analysis considering all the four diagnostic processes. 

Tongue diagnosis is a very important diagnostic procedure in TKM, despite its inevitable limitations associated with clinician experience and knowledge. However, this study shows that interobserver reliability in tongue status in stroke patients between the raters was considerably high. This may help to alleviate the lack of objectivity and reproducibility in tongue diagnosis in TKM. We expect that future studies will help to further establish tongue diagnosis as a useful oriental medicine diagnostic tool in the clinical management of stroke patients.

## Figures and Tables

**Table 1:** Agreement between raters for all subjects.

Variables	% Agreement	Kappa (*κ*)	CI of *κ*	AC_1_	CI of AC_1_
Tongue color:					
Pale	71.38	0.42	(0.35, 0.49)	0.43	(0.36, 0.51)
Red	75.84	0.51	(0.44, 0.58)	0.52	(0.45, 0.59)
Bluish purple	91.99	0.42	(0.35, 0.49)	0.9	(0.87, 0.93)
Fur color:					
White fur	75.17	0.49	(0.42, 0.56)	0.51	(0.44, 0.59)
Yellow fur	85.29	0.69	(0.63, 0.75)	0.71	(0.66, 0.78)
Fur quality:					
Thick fur	81.15	0.60	(0.54, 0.67)	0.63	(0.57, 0.70)
Thin fur	74.61	0.49	(0.41, 0.56)	0.49	(0.42, 0.57)
Moist fur	70.27	0.29	(0.21, 0.37)	0.49	(0.42, 0.57)
Dry fur	80.5	0.48	(0.40, 0.56)	0.68	(0.63, 0.75)
Special tongue appearance:					
Teeth marked	87.68	0.46	(0.36, 0.56)	0.84	(0.80, 0.88)
Enlarged	89.63	0.51	(0.41, 0.61)	0.86	(0.83, 0.90)
Mirror	97.44	0.60	(0.42, 0.78)	0.97	(0.95, 0.99)
Spotted	96.96	0.37	(0.15, 0.59)	0.96	(0.95, 0.98)

CI: confidence interval.

**Table 2:** Agreement measures in PI with the same opinions between the raters.

Variables	% Agreement	Kappa (*κ*)	CI of *κ*	AC_1_	CI of AC_1_
Tongue color:					
Pale	75.00	0.49	(0.41, 0.58)	0.51	(0.42, 0.59)
Red	76.67	0.53	(0.45, 0.61)	0.54	(0.46, 0.62)
bluish purple	94.09	0.57	(0.42, 0.72)	0.93	(0.90, 0.96)
Fur color:					
White fur	77.05	0.52	(0.44, 0.60)	0.56	(0.48, 0.64)
Yellow fur	86.37	0.71	(0.64, 0.78)	0.74	(0.68, 0.81)
Fur quality:					
Thick fur	83.25	0.65	(0.58, 0.73)	0.68	(0.61, 0.75)
Thin fur	75.18	0.50	(0.42, 0.58)	0.51	(0.42, 0.59)
Moist fur	70.53	0.31	(0.22, 0.40)	0.5	(0.41, 0.58)
Dry fur	82.46	0.52	(0.42, 0.61)	0.72	(0.66, 0.79)
Special tongue appearance:					
Teeth marked	89.06	0.53	(0.42, 0.64)	0.86	(0.82, 0.90)
Enlarged	90.67	0.57	(0.46, 0.69)	0.88	(0.84, 0.92)
Mirror	97.99	0.72	(0.54, 0.89)	0.98	(0.96, 0.99)
Spotted	96.88	0.40	(0.15, 0.65)	0.97	(0.95, 0.99)

PI: pattern identification; CI: confidence interval.
